# Assessment of Biodiversity in Food Consumption Studies: A Systematic Review

**DOI:** 10.3389/fnut.2022.832288

**Published:** 2022-06-14

**Authors:** Maria Fernanda Araújo de Medeiros, Stephanie Gomes Bezerra Silva, Carla Djaine Teixeira, Severina Carla Vieira Cunha Lima, Dirce Maria Marchioni, Michelle Cristine Medeiros Jacob

**Affiliations:** ^1^Nutrition Department, Federal University of Rio Grande do Norte, Natal, Brazil; ^2^Graduate Program in Social Sciences, Center for Human Sciences, Letters and Arts, Federal University of Rio Grande do Norte, Natal, Brazil; ^3^Nutrition Department, School of Public Health, University of São Paulo, São Paulo, Brazil

**Keywords:** food biodiversity, food consumption, food security, sustainable development goals, ethnonutrition

## Abstract

The assessment of food biodiversity has gained importance in nutrition due to the positive association between the diversity of foods consumed and the quality of diets. To date, however, we do not know systematically how food consumption studies address food biodiversity. Our objective with this paper was to characterize how food consumption studies address biodiverse foods, both in terms of (i) new methods capable of overcoming the limitations of existing methods, and (ii) indicators capable of measuring the contribution of biodiversity to nutrition. We conducted a systematic review based on the Preferred Reporting Items for Systematic Reviews and Meta-Analysis (PRISMA), using four databases: Web of Science, Medline/PubMed (*via* National Library of Medicine), Scopus, and Google Scholar. We selected papers focused on the consumption of biodiverse foods without time constraints. In addition, we assessed the methodological quality of the studies we selected. We reviewed a total of 22 studies, and summarized the methods and indicators most used. We found that some researchers used biodiversity mapping strategies based on ethnographic approaches before the dietary assessment. Regarding dietary assessment tools, retrospective direct methods were the most used by researchers. We list 23 indicators used by the authors, among them the Dietary Species Richness (DSR), used in 18% of the studies. Studies that used biodiversity mapping strategies based on ethnographic approaches before the dietary assessment portrayed the local availability of biodiverse foods more consistently, i.e., presented lists with local edible species satisfactorily identified. We believe researchers in the future can avoid many of the limitations of current methods by ensuring that teams are interprofessional. We emphasize that most of the indicators we summarized are not sensitive enough to biodiversity since they do not measure edible resources at the species level. In this sense, the DSR is promising, because it fills information gaps, especially in the case of wild or neglected species.

## Introduction

Biodiversity is the biological diversity of animals, plants, fungi, algae, and other organisms, including diversity within species, among species, and within ecosystems (1). The term biodiverse foods refers to the subset of these resources that are edible and available in a food system. This definition encompasses cultivars and varieties of conventional foods (e.g., types of beans, local chicken breeds), as well as species considered as non-conventional or of limited cultural use, also referred to as wild, native, neglected, and spontaneous species (2, 3).

In recent years, the assessment of food biodiversity consumption has gained importance in nutrition due to the positive association between the diversity of foods consumed and the quality of diet. For example, Lachat et al. (4) analyzed the contribution of diversity within food consumption of women and children (*n* = 6,226) in rural areas of seven low- and middle-income countries. They found a positive association between dietary species richness, or the count of the number of different species consumed per day, and the nutritional adequacy of diets. Besides, it is worth mentioning that the benefits related to food biodiversity are not limited to human health outcomes. Nowadays, we have the opportunity to address several global challenges related to nutrition by diversifying diets. Some of these challenges include meeting high per capita demand for nutrient-rich foods (5), fostering resilience to climate change and to the emergence of new zoonotic outbreaks, and promoting stability in food supply within the food system (6). One of the most comprehensive reviews to date examining the role of biodiversity in sustainable development highlights that biological diversity can directly contribute to (i) increased food and nutrition security levels due to a rise in local food consumption; (ii) reduced poverty because local consumption generates a source of income for local farmers; (iii) improved health and wellbeing due to higher access to more nutritious foods; and (iv) increased ability to mitigate climate change by considering as food those species adapted to local ecosystems (1). Therefore, biodiversity not only contributes to the nutritional quality of diets, but it also fosters planetary health.

Despite the strategic role of biodiversity to promote human and environmental health, food consumption studies still have a narrow approach to the topic due to two main limitations: lack of food composition data and lack of appropriate food consumption assessment tools for mapping biodiverse foods in current food systems (3). First, regarding composition data, we know that many food composition tables do not present satisfactorily the edible biodiversity of their countries of origin, which leads to misinterpretations in the assessment of diets, which consequently can lead to inefficient nutrition policies, such as supplementation or fortification programs (7). Furthermore, by analyzing food at the taxonomic level below species, we know that the nutrient content and bioactive compounds can vary significantly among different varieties or cultivars of the same species (8). Burlingame et al. (9) present valuable examples of how high this variation can be considering species of conventional plants, such as rice, potatoes, mangoes, and bananas. For example, considering the content of carotenoids, they observe that some bananas may have up to 8,500 times more beta carotene when compared to other varieties. Furthermore, recent studies have highlighted the nutritional relevance of wild or native species, showing that even though the contribution of these neglected species to energy content of diets seems to be insignificant, they are a relevant source of several micronutrients of global nutritional interest, such as iron, zinc, vitamin A, and folate (10). Second, regarding dietary assessment methods, we know that many research protocols have limitations in addressing food diversity due to inadequate cultural adaptation of the survey tools (3). The lack of cultural adaptation of dietary assessment tools can led to two major mistakes: first, to over- or underestimate energy, macro- and micronutrients, bioactive compounds, and anti-nutritional factors in dietary assessments; second, to ignore under- or over-reporting of some food resources in dietary inquiries (11). This weakness of dietary assessment tools compromises our capacity to perceive the presence of local varieties in diets and thereby limits our ability to analyze the nutritional relevance of the species and their varieties to food and nutrition security (9).

In recent years, however, the assessment of local biodiversity has started to play a part in food consumption studies, especially those with a focus on species considered native, indigenous, wild, and traditional (12–15). However, to date, we do not know systematically how these assessments address food biodiversity, both in terms of (i) new methods capable of overcoming the limitations of existing methods, and (ii) indicators capable of measuring the contribution of biodiversity to nutrition. Therefore, with this systematic review, we seek to answer the following question: “What are the methods and indicators used in the assessment of biodiverse foods in food consumption studies?”

## Methods

We conducted a systematic review based on the Preferred Reporting Items for Systematic Reviews and Meta-Analysis (PRISMA) Statement (16)—Supplementary File 1. Our protocol for this review was not previously registered because our research does not analyze directly any health-related outcomes.

### Selection Criteria

We selected articles following these eligibility criteria: (i) original articles, published in English, Spanish, or Portuguese; (ii) papers focused on the assessment of food consumption of human populations; (iii) research presenting outcomes related to the consumption of biodiverse foods; and, finally, (iv) our investigation considered papers without time constraints. We excluded (i) repeated articles and (ii) review products.

### Search Sources and Strategy

During March 2021, MFAM performed the search using four databases: Web of Science, Medline/PubMed (*via* National Library of Medicine), Scopus, and Google Scholar. The search consisted of applying the descriptors in each database. The entire search strategy is available in Supplementary File 2.

### Study Selection

With the assistance of the tool Mendeley, MFAM organized all records and deleted duplicates. By applying the eligibility criteria previously outlined, two authors (MM and SS) selected the articles individually. Initially, titles and abstracts underwent a first screening, at which point we excluded those that did not meet the selection criteria. In cases of discrepancies or uncertainties about inclusion, we consulted a third author (MJ). Then, we proceeded to a full reading of potentially eligible texts.

### Data Extraction

Three authors (MFAM, SGBS, and CDT) extracted data from the selected articles into a spreadsheet designed to assist us in answering the research question. Next, MCMJ verified the accuracy and scope. We gathered the following information: (i) article data (authors, year of publication, and journal), (ii) research setting and number of participants, (iii) research design, (iv) objective, (v) biodiverse foods assessed (e.g., wild plants, cultivated plants, bushmeat, mushrooms), (vi) methods applied to assess biodiversity, (vii) indicators, (viii) main results, (ix) main limitations reported, and (x) quality.

### Quality Analysis

We evaluated the methodological quality of the studies by adapting a consolidated protocol to the objectives of our study (17). The consolidated protocol chosen, considering the design of the studies we reviewed, was the STROBE (Strengthening the Reporting of Observational Studies in Epidemiology Statement). STROBE consists of a checklist of 22 essential items applied to observational epidemiological studies. Considering the specificity of our analysis, and the absence of more specific protocols, we added three new items to the checklist in order to evaluate (i) whether the research team mapped foods species previously to the dietary assessment, (ii) if the authors report having checked the taxonomy of species, and (iii) whether the paper specifies environmental conditions when characterizing the setting (e.g., climate, soil, etc.) due to the importance of this information to biodiversity analysis. The adapted instrument contains 25 items.

After analyzing all the items, the studies received a point for each criterion fulfilled. Based on the grades received, we used three categories for quality assessment: strong—when the study met more than 80% of the criteria; moderate—from 50 to 80%; weak— <50% (18).

### Summary of Results

We synthesized results by producing narrative summaries of each of the articles eligible for a full reading. During the reading process, we focused on detecting (i) methods used in the assessment of biodiverse foods and (ii) indicators employed to measure the contribution of biodiversity to nutrition reported in the manuscripts.

To present our results more clearly, we grouped the methods and indicators in the following ways. We grouped the methods into those applied (i) to map then classify known and consumed species and (ii) to assess food consumption. As for the indicators, we grouped them into four levels of analysis ranging from the landscape to the nutrient level: (i) production, collection, or supply: indicators that measure the number of species and varieties found within a given area, sample, farm, or local market; (ii) food consumption by population or household: indicators that refer to the number of food groups or food items consumed in a given population or household; (iii) food consumption by individuals: indicators that refer to the number of food groups or food items consumed by a person; and, finally, at the most specific level, (iv) dietary intake by individuals: indicators that measure individual food consumption at the nutrient level.

## Results

### Study Selection

**Figure 1:**
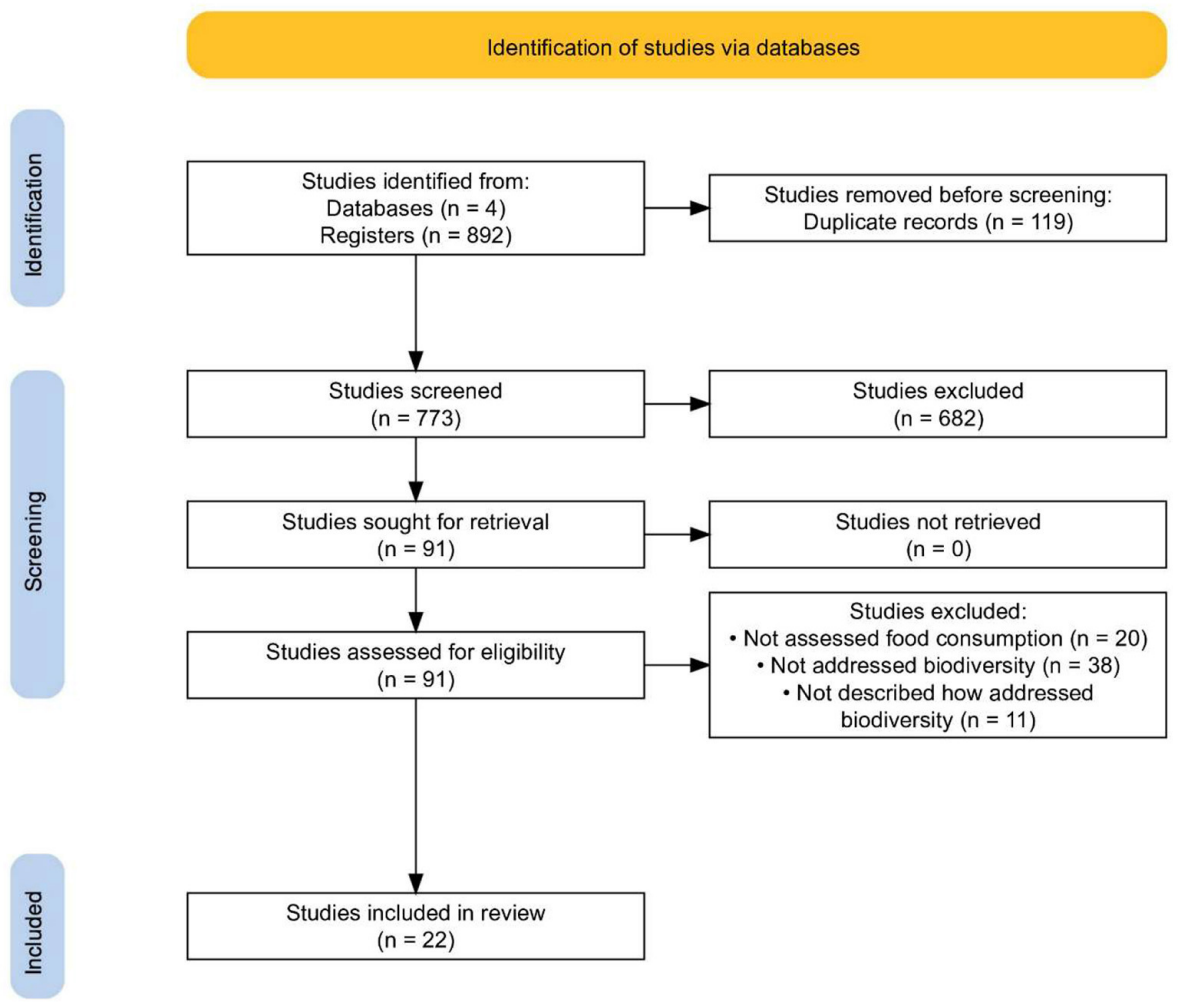
Flowchart of the study selection process.

The search in the databases led to the recovery of 892 studies (138 in the Web of Science, 112 in Medline/PubMed, 401 in Scopus, and 241 in Google Scholar). After excluding 119 duplicates, we considered 773 articles as eligible for the next stage of selection. Based on titles and abstracts, we selected 91 papers for a full reading. At this stage, we excluded 69 articles that did not fit our inclusion criteria. Therefore, a total of 22 articles make up this review. Figure 1 shows the study selection process and the related flowchart.

### Study Characteristics and Quality

Table 1 provides an overview of the main characteristics of the 22 studies included in this review.

**Table 1:** Characterization of food consumption studies assessing biodiverse foods.

**Study number**	**Article data**	**Research setting and number of participants**	**Research design**	**Objective**	**Biodiverse foods assessed**	**Methods applied to assess biodiversity**	**Indicators**	**Main results**	**Main limitations reported**	**Quality**
1	Blundo-Canto et al. (19)	Ucayali, Peru, *n* = 53 households in 4 rural communities	Observational, longitudinal	Test the hypothesis that the expansion of commercial crops is associated with deforestation, reduced agrobiodiversity, and food access changes	All foods, including bushmeat and species of local plants	Use of one 24-h recall in 2000 and another in 2015. Besides 24-h recall in 2015, the analysis combined in-depth interviews and focus groups with key informants in order to evaluate consumption changes	Household dietary diversity score (HDDS)	Regarding food consumption, the authors observed a reduction in the household food diversity score (HDDS) of 1.3 food groups when comparing data from 2000 and 2015. In addition, the variety of food items consumed also decreased from 69 food items mentioned in 2000 to 35 in 2015	Researchers used respondents' recall and perception to build their conclusions instead of empirical quantification	Strong
2	Boedecker et al. (20)	Benin, *n* = 120 households, non-pregnant and non-lactating women (>18 y), rural community	Observational, cross-sectional	To evaluate the contribution of wild food plants within the diets of women living in the buffer zone around Lama forest, southern Benin	Wild edible plants	The authors evaluated the consumption of wild plants with two non-consecutive 24-h recall. They conducted the taxonomic identification of some species in the field. The species they were unable to identify were collected (dry specimens and photos) and taken to the herbarium	Women Dietary Diversity Score (WDDS) and Nutrient Adequacy Ratio (NAR, considering EAR)	The contribution of WEPs to total dietary intake was low due to infrequent use and small portion sizes. The highest nutrient contributions of WEPs measured were for copper (13.9%) and iron (4.6%). Women's dietary diversity was significantly higher among WEP consumers than non-consumers, mainly due to higher consumption of dark green leafy vegetables	Some plants cited by participants were not available due to seasonality, while others were inaccessible due to legal restrictions in the forest	Strong
3	Broegaard et al. (21)	Laos, *n* = 33 households in 3 rural communities	Observational, cross-sectional	Examine the role of agricultural and forest landscapes in providing wild foods in farming communities in northern Laos	Wild foods characterized as sources of protein in the local context, e.g., rice, rat, and fish	Use of field collection diaries in four agricultural cycles (i.e., slashing and burning, planting, weeding, and harvesting) to ensure variation in different seasons. Research assistants identified all hunted animals to the taxonomic group rather than at the species level. Researchers conducted interviews and participant observation, but there is no mention of classic dietary assessment methods	Proportion of protein from wild food in relation to the per capita value of 50 g of protein per day	Increased land use pressure alters the cultivation landscape and, consequently, the quality of the diet, with adverse effects on protein intake. Wild plants contributed much more to a diversified diet in villages with traditional crop farming systems and much less in villages dominated by commercial crop production	Participants may have (intentionally) failed to report or display all collected products, especially the illegal ones. Authors report that it was difficult to estimate protein contributions from wild foods due to lack of composition data	Moderate
4	Chyne et al. (22)	Meghalaya, India, *n* = 160 households in 15 rural communities comprising *n* = 510 (women and children <5 y)	Observational, cross-sectional	Examine whether the prevalence of malnutrition and chronic disease among the Khasis of North East India is associated with available food biodiversity	All food sources	The authors applied a 24-h recall to assess consumption. To evaluate the knowledge about food plants (cultivated or wild), they conducted free listing and focus groups. However, researchers did not identify the taxonomy of the species consumed; they just recorded local names	Average consumption of nutrients compared to Recommended Dietary Allowances (RDA) and Species consumed by food groups (number without scientific identification)	Nutrient intake was below recommended levels. The prevalence of anemia in children aged 1–5 was 68%, and vitamin A deficiency was 59%, and among women, the same number ranged between 83 and 48%, respectively. Through focus groups, villages reported using 372 food species, most of which were wild (225). According to the authors, malnutrition is unacceptably high among the Khasis, considering the richness of food biodiversity	Due to the lack of nutrient data for many local foods, the numbers of nutrient intake are just estimations. Furthermore, the authors did not perform taxonomic identifications for more than two-thirds of the food species	Strong
5	Conti et al. (15)	Arusha, Tanzania, *n* = 141 households in urban and peri-urban areas, women of reproductive age (15–4 9y)	Observational, cross-sectional	Explore aspects that contribute to micronutrient adequacy in women of reproductive age in Tanzania, focusing on indigenous vegetable consumption and other sociodemographic factors	Indigenous vegetables, i.e., neglected or underutilized plants	Use of a 24-h recall applied by trained interviewers, supported by a photo guide to assess portion sizes. Researchers used a list of edible resources elaborated in a previous study to identify the vegetables mentioned in the 24-h recall	Minimum dietary diversity index for women (MDD-W)	Results showed that sixteen percent of the women consumed at least one serving of indigenous vegetables per day. Indigenous vegetable consumption was positively associated with micronutrient adequacy	In statistical terms, the women interviewed in the survey did not represent the Arusha region and Tanzania, considering demographic, social, educational, and economic variables. In addition, researchers did not assess rural areas and the data gathering just covered the dry period	Strong
6	Da Silva and Begossi (23)	Amazon, Brazil, *n* = 114 households in the urban area of Barcelos and in 3 rural communities	Observational, cross-sectional with a longitudinal component to assess seasonality in food consumption	Describe the food consumption of riverine populations, comparing the composition, origin, diversification, and seasonal variations in diets of different communities	Animals: fish, birds, and mammals	Researchers performed data collection both in dry and rainy seasons, with the support of the 24-h recall and participant observation. The research team identified the taxonomy of fish samples by comparison in collections of research institutes, birds and mammals, by using field guides; and, finally, experts reviewed mammals identification	Shannon-Wiener Diversity Index (H′) and Species Richness	About 80 species of consumed fish were collected and identified in the studied populations. Fish account for 70% of the protein in the main meal, being unusual the consumption of beef. Families in the rural setting, and with low-income, had lower species richness in their diets. Industrialized and imported animal protein products (e.g., sausage, beef, frozen chicken, dairy products, powdered milk) account for 134 items consumed among urban households vs. 13 items among rural households. The consumption of imported items increased species richness, both in urban and rural areas	There is no report	Moderate
7	Fungo et al. (24)	Cameroon, *n* = 279 households in 12 rural communities, women	Observational, cross-sectional	Determine the contribution of forest foods to diets and estimate their association with household food insecurity	Forest plants	Use of two 24-h recall with an interval of 1 week. Researchers conducted focus groups in order to build a list of forest plants consumed by the population. Plant specimens were photographed, collected, and taken to the herbarium to be identified	Household dietary diversity score (HDDS), Food variety score (FVS), Forest food consumption score (FFCS), Household food insecurity access scale (HFIAS), and contribution to nutrient intake (NAR/EAR)	Researchers identified 47 forest plants. Of these plants, 17 were consumed by 98% of respondents over a week. Forest foods contributed approximately half of women's total daily energy intake in the case of women. The most significant contributions were for intakes of vitamin A (93%), Na (100%), Fe (85%), Zn (88%), and Ca (89%). Despite the high biodiversity, most families (83%) suffered from high food insecurity. Results shown that forest foods play an essential role in ensuring food security in these forest-dependent communities	There is no report	Strong
8	Ghosh-Jerath et al. (25)	Gumla, Jharkhand, India, *n* = 143 households in 4 rural tribal communities, women of reproductive age	Observational, cross-sectional with a longitudinal component to assess seasonality in food consumption	Assess the availability and consumption of indigenous food plants and the nutritional status of women from the Oraon tribal community in Jharkhand, India	Indigenous food plants, i.e., those obtained locally through cultivation or collection.	Use of 24-h recall on two consecutive days in winter, summer, and monsoon season. In winter, researchers also applied the FFQ. FFQ items, which included conventional and indigenous foods, were identified in focus group discussions. The authors mention in their acknowledgments that they developed the taxonomic classification of species samples	Nutrient adequacy ratio (NAR, RDA) and Household food security score	Food security and consumption of indigenous foods were low. Although the community reported knowing various indigenous foods (244 food items), the study revealed that the regular consumption of these items was insufficient. Higher intake of essential micronutrients, calcium, and iron, was observed among those who consumed indigenous foods. About 40% of women had degrees of chronic energy deficiency	Researchers calculate energy requirements by considering moderate physical activity levels for women. However, they believe that some women could have higher physical activity levels because activities such as cutting firewood, working in brickyards, herding cattle are common in these communities. These underestimations of energy may explain the higher prevalence of chronic energy deficiency observed, despite a caloric intake close to 80% of recommended levels. In addition, 24-h recall of two consecutive days was conducted in only one-third of the study sample	Strong
9	Ghosh-Jerath et al. (26)	Gumla, Jharkhand, India, *n* = 151 households in 4 rural tribal communities, women of reproductive age	Observational, cross-sectional with a longitudinal component to assess seasonality in food consumption	Explore the contribution of indigenous foods to nutritional status and nutrient intake	Indigenous food plants, i.e., those obtained locally through cultivation or collection	Use of 24-h recall on two consecutive days during the rainy season reproduced in winter and summer; FFQ was applied in the rainy season. Researchers conducted qualitative surveys (e.g., key informant interviews and focus group discussions) to capture the variety of foods consumed by the community. The researchers classified these species by their taxonomy	Nutrient adequacy ratio (NAR, RDA) and Household food security scale	The communities reported knowing a wide variety of indigenous food plants even though they did not consume them. Women consumed adequate energy and protein, but micronutrient intake was inadequate. For example, consumption of Ca, Fe, vitamin B2, folate, and vitamin B12 was insufficient in more than half of the participants. Women who consumed indigenous foods during the survey period had significantly higher intakes of Ca and Fe when compared to those who did not consume	The 24-h recall for two consecutive days may overestimate the prevalence of inadequacy of micronutrients in the population. Researchers did not analyze the composition of three foods items	Strong
10	Ghosh-Jerath et al. (27)	Gumla, Jharkhand, India, *n* = 204 households in 18 rural tribal communities, women of reproductive age	Observational, cross-sectional with a longitudinal component to assess seasonality in food consumption	Explore the association between production and access to agroforestry foods and consumption of indigenous plants with nutrient adequacy in the Sauria Paharia community	Indigenous food plants, i.e., those obtained locally through cultivation or collection	Use of 24-h recall on two consecutive days during the rainy season, repeated in winter and summer; FFQ was applied in the rainy season. Researchers conducted qualitative surveys (e.g., key informant interviews and focus group discussions) to capture the variety of foods consumed by the community. The researchers classified these species by their taxonomy	Food Accessed Diversity Index (FADI) and Median minimum dietary diversity score for women (MDD-W)	Access to agroforestry diversity was low (low FADI), despite the extensive knowledge of people about local plants. Women with the highest dietary diversity score (MDD-W) had higher intakes of energy, protein, fat, iron, calcium, zinc, B vitamins, vitamin A, and vitamin C. In addition, consumers of indigenous plants had higher intakes of calcium and vitamin A	Researchers calculated nutrient intake using software that provides the nutritional value of raw Indian foods. The FFQ was too long (300 items), which could lead to reporting and recall bias	Strong
11	Golden et al. (28)	Madagascar, *n* = 719 individuals (0–73 y), 152 households, in 2 rural communities	Observational, longitudinal	To characterize the consumption patterns of the Malagasy, living in remote rainforest areas in northeastern Madagascar	All food sources, including bushmeat and native plants	Weighed Food Record of three meals a day during nine consecutive months. Considering the information provided in the paper, researchers did not classify species by their taxonomy	Mean Adequacy Ratio (MAR, EAR), Household dietary diversity score (HDDS), Food consumption score (FCS), and Minimum dietary diversity for women (MDD-W)	Although the HDDS and FCS reflect the high diversity of the diet, the MDD-W indicator suggests poor micronutrient adequacy. For example, the median individual consumed <50% of their average requirement (EAR) for Ca and vitamins A, B12, D, and E; and <100% of their EAR for energy, riboflavin, folate, and Na	Lack of adequate nutrient composition data for many locally endemic foods, driving the authors to use established proxies that may not accurately represent the nutrient content of local foods	Moderate
12	Jones (29)	Malawi, *n* = 3,000 households of agricultural families, national	Observational, longitudinal	Determine the association of cultivated species richness with the diversity and quality of family diets in Malawi and assess the hypothetical mechanisms for this association through livelihood and market-oriented pathways	Cultivated plants, including market and subsistence crops	7-day recall. Considering the information provided in the paper, researchers did not classify species by their taxonomy	Crop species richness (CSR) and Diet diversity score (DDS)	Agricultural biodiversity was a key determinant of the diversity and quality of the diets of farming families in Malawi. Neither the proportion of the harvest sold nor the distance to the nearest population center changed the relationship between CSR and DDS. Families with higher CSR were more commercially oriented	The DDS calculation was based on 7-day recall data at the household level. Given the extended recall period and data aggregation at the household level, the DDS was high for most households. Although the DDS was correlated with energy and nutrient intake, a more discerning indicator based on individual 24-h recall data would likely serve as a better metric. Second, food composition tables may be limited in their ability to accurately quantify the nutrient composition of food items from distinct agroecological contexts	Strong
13	Jones et al. (30)	Andes, Peru, *n* = 600 households in 48 different communities, women of reproductive age	Observational, cross-sectional	To determine the association of agricultural biodiversity within agricultural properties with the diversity and quality of the diet among women of reproductive age in Peru, evaluating the effect of the market on this association	Cultivated plants and animal husbandry (poultry, goats, and sheep)	Use of a 24-h recall, with a repeat in a sample of 100 women. Considering the information provided in the paper, researchers did not classify species by their taxonomy	Dietary species richness (DSR), Diet diversity score (DDS), Minimum dietary diversity for women (MDD-W), Probability of adequacy (PA), and Crop species richness (CSR)	Agricultural biodiversity within farms was associated with moderately more diversified and more micronutrient-rich diets among Peruvian women. Agricultural market orientation did not mediate these associations	With an observational design, the ability to draw a causal inference from the observed associations is limited. The recall period (i.e., 24 h) for the independent variable (i.e., agricultural diversity in the prior agricultural season) did not fully align, which may lead to underestimating the association between farm biodiversity and diet outcomes. There are also limitations in the data from the food composition table available, which made it impossible to calculate the probable intakes of vitamins B6 and B12	Strong
14	Kennedy et al. (31)	Saturia, Bangladesh, *n* = 313 households in 10 rural communities	Observational, cross-sectional	Describe the consumption of plant genetic diversity	Cultivars of food plants (rice, pulses, eggplant, potatoes, and bananas)	Researchers used various qualitative research techniques to adapt indicators to the local context, such as interviews with key informants, free listing, market research, and participant observation. They also adapted the 24-h recall to include indicators of genetic diversity and applied it to women in the households	Plant genetic diversity (PGD) of cultivars and germplasms	Using these two indicators in the context of using 24-h recall was an initial attempt to classify crop diversity. Women interviewed were able to identify many of the cultivars consumed. This degree of agricultural knowledge would likely decrease in urban settings or areas where agriculture is not the primary occupation. The authors also found that girls are a fundamental source of knowledge about green leafy plant species, as they are primarily responsible for collecting these vegetables. In-depth qualitative research before the dietary survey is essential for developing food codes in order to achieve a reasonable level of precision when applying the indicators	There is no report	Moderate
15	Lachat et al. (4)	Diversos países: Benin, Cameroon, Congo, Ecuador, Kenya, Sri Lanka, Vietnam, *n* = 6,226 (2,188 women e 4,038 children), rural communities	Observational, cross-sectional	Test different indicators for diets assessment in order to recommend a cross-cutting indicator capable of measuring biodiversity in the human diet, guiding interventions for human, and environmental health simultaneously	All food sources	Secondary data from the application of one 24-h recall (*n* = 6,226) between 2009 and 2015	Dietary species richness (DSR), Diet diversity score (DDS), Simpson Diversity Index (D), Functional diversity (FD), and Mean adequacy ratio (MAR)	People consumed 234 different species, of which <30% were eaten in more than one country. Nine species were consumed in all countries and provided, on average, 61% of the total energy consumption and a significant contribution of micronutrients in the rainy season. Comparing indicators, the DSR was the one that best predicted diet quality. For each additional species consumed, the adequacy of dietary nutrients increased by about 3%. Therefore, the authors recommended DSR as the EST indicator to assess food biodiversity in diets	They used a single 24-h recall per subject. This method does not allow accounting for within-person variability and estimation of usual dietary intake. During the analysis, there was a lack of nutrient composition data for some foods, species, and varieties consumed	Moderate
16	M'Kaibi et al. (32)	Kenya, *n* = 525 households in 2 rural communities, children (24–59 m)	Observational, cross-sectional with a longitudinal component to assess seasonality in food consumption	Assess the effects of agricultural biodiversity and seasonal rains on the adequacy of diet and food security of families of preschool children	Whole diet, focusing on animals (hunted and farm-raised) and other food items obtained in natural habitats through hunting or gathering	The authors conducted a pre-dietary survey assessment to map local agrobiodiversity, including interviews with key local informants (elders), and focus group discussions. They applied four 24-h recall per person, two non-consecutive in the dry season and two in the rainy season	Household food insecurity access scale (HFIAS), Nutrient adequacy ratio (NAR), and Mean adequacy ratio (MAR)	Food intake was low, with most households not meeting the RDA for many nutrients. However, intake of energy, protein, Fe, Zn, Ca, and folate significantly improved in the rainy season. Agricultural biodiversity was positively related to all NARs and MAR, indicating a significant positive relationship between household agricultural biodiversity and children nutrition	The two areas studied were not so similar in terms of their agricultural and physical resources, despite being physically very close. The authors evaluated only foods grown or obtained in nature when assessing agricultural biodiversity, excluding foods purchased in stores and markets	Strong
17	Mathewos et al. (33)	Yayo Reserve, Ethiopia, *n* = 96 households in 4 rural communities	Observational, cross-sectional	Assess the contribution of fruit and vegetables grown in backyards to the livelihood of families and conservation of biodiversity	Fruits and vegetables produced in domestic kitchen gardens	The authors produced the biodiversity inventory through the assessment of 48 home gardens, considering plant diversity, frequency, and density. Researchers classified plants by their taxonomy. To assess the perceived contribution of fruits and vegetables to the livelihoods of family farmers, the researchers conducted semi-structured questionnaires, focus group discussions, and direct observation. The research did not use recognized dietary assessment methods	Shannon-Wiener Diversity Index (H′)	The size of the home gardens had a positive association with species richness. The authors demonstrated that fruits and vegetables in home gardens contributed considerably to the diets of families. The result revealed the direct and indirect contribution of fruits and vegetables in home gardens to biodiversity conservation. The direct contribution is that the species of fruits and vegetables grown in their backyards contributes to the increase of plant diversity. The indirect role is thar fruits and vegetables in the home gardens reduced the use pressure of forest products	There is no report	Moderate
18	Ntwenya et al. (34)	Kilosa District, Morogoro Region, Tanzania, *n* = 307 households in 3 rural communities	Observational, cross-sectional with a longitudinal component to assess seasonality in food consumption	Describe the foods available and consumed in the Kilosa district of Tanzania	All food sources	The researchers listed edible resources available before conducting the dietary survey by consulting members of local communities and by conducting market surveys. They classified plants by their taxonomy with the help of a botanist. Then, they performed a dietary assessment by using a 24-h recall in both stations. Different parts, forms, or stages of ripeness of the same food reported were counted separately	Food biodiversity score (FBS)	A total of 183 edible food items were reported by households, with more reports in the rainy season (*n* = 82) than in the harvest season (*n* = 64). The average number of foods consumed per day during the rainy season was significantly higher than in the harvest season. About 50% of families mentioned that their family members had a low acceptance of wild edible foods	There is no report	Moderate
19	Penafiel et al. (14)	Andes, Guasaganda, Ecuador, *n* = 178 households in 10 rural communities, indigenous women	Observational, cross-sectional	Assess dietary diversity and nutrient contribution of traditional foods	Traditional plants, i.e., grown locally or wild	Use of two 24-h recall with an interval of 14 days between applications during the rainy season. Afterward, wild species were collected with the help of a local guide and identified by their taxonomy. Researchers also used a FFQ was used to estimate the frequency of consumption	Nutrient adequacy ratio (NAR/EAR), Mean adequacy ratio (MAR), Dietary species richness (DSR), Traditional food diversity score (TFDS) and Minimum dietary diversity for women (MDD-W)	Researchers found a positive association between the consumption of traditional foods and the adequate intake of macro and micronutrients. The average diet had a MAR of 0.78. The consumption of traditional foods contributed 38.6% of the total energy intake. Higher consumption of local species was associated with an increase in the median MAR of macronutrients of 3.3% and micronutrients of 5.2%	The research team did not reach all households during one of the research stages due to torrential rain that limited access to some families. Due to legal issues, some animal samples were not collected	Strong
20	Remans et al. (35)	Malawi, Kenya, and Uganda (Sub-Saharan Africa), *n* = 170 households, in 3 rural communities	Observational, cross-sectional	Explore how functional nutritional diversity indicators can provide insights into the nutrient diversity of agricultural systems	Food plants	Use of a 24-h recall. Researchers classified species by their taxonomy, comparing samples with studies of the local flora	Functional diversity (FD): total, macronutrients, minerals, and vitamins.; Household food security access scale (HFIAS); Months of inadequate household food provisioning (MIHFP); and Household diet diversity scores (HHDDS)	Researchers identified a total of 77 species of edible plants. The application of FD allowed the identification of key species that add nutrient diversity to the system. The analysis has shown that adding or removing individual species can radically alter nutritional diversity. The authors advocate the use of this indicator as a tool capable of relating inputs from agriculture, human nutrition, and ecology	Researchers did not collect data on the quantities of food plants produced or the uniformity of these species. In addition, composition data did not include foods below the species level	Moderate
21	Termote et al. (12)	Congo, *n* = 492 women, rural (129) and urban areas (363) from different cities	Observational, cross-sectional	Describe the contribution of wild plants to local diets	Wild food plants	In previous ethnobotany research, researchers collected and identified species. They assess food consumption by using two non-consecutive 24-h recall	Nutrient adequacy ratio (NAR, by RDA)	The results showed that wild plants are insufficiently consumed in a biodiversity hotspot and precarious food security. The most significant contribution came from Dacryodes edulis, contributing 4.8% of the total energy intake. Considering the nutrient composition of the various wild plants available in the region and known to indigenous peoples, the potential for increasing food security is vast. Researchers argue that ethnobiological research must find ways to separate knowledge from the consumption of plant species	There was no information on the nutritional composition of these foods. Democratic Republic of Congo at the time did not have a food composition table	Strong
22	Wertheim-Heck and Raneri (36)	Hanoi, Vietnam, *n* = 389 households, women of reproductive age, urban area	Observational, mixed methods	Test whether the diversified retail offer contributes to more diversified and nutritionally balanced diets	All food sources	The study began by mapping retail food outlets within the study area. Next, the household consumption survey used the 24-h recall, with a sub-sample (*n* = 60) of non-consecutive application, considering species and varieties. Considering the information provided in the paper, researchers did not classify species by their taxonomy. In the second phase of the study, the authors conducted interviews and shopping trips; and finally, developed a documentary film with three participants detailing food purchase and consumption practices at home	Diet diversity score (DDS), Minimum diet diversity (MDD), Mean adequacy ratio (MAR), and Nutrient adequacy ratio (NAR)	The study found that supermarkets and convenience stores offer a higher percentage and a more extensive range of ultra-processed products than traditional open-air markets. Furthermore, the authors state that traditional establishments (e.g., wet markets, street markets, and market stalls) were essential for maintaining minimum dietary adequacy for poor people living in cities. However, in statistical terms, the diet quality in the different strata was not influenced by the geographic proximity of formal points of sale. On average, the MAR was just 0.54, which means that the women consumed just over half of their daily nutrient requirements	A limitation of the study design is the large response load on participants, generated through several follow-up surveys. Another limitation was the repetition of the 24-h recall with an interval of 1 year	Moderate

The earliest study included in this review dates back to 2005 (31). Of the 22 papers, 20 were published in the last 10 years, with 73% from 2016 onwards. All studies have an observational design: 18 cross-sectional (six have a longitudinal component to assess seasonality in food consumption), three longitudinal, and one mixed methods (ethnographic and food consumption approaches).

We found the quality of studies to be either moderate or strong. Characteristics that most contributed to a moderate rating were: not mapping edible species prior to dietary assessment; not reporting taxonomic classifications of species; lack of precision in reporting numbers of participants in each stage of the study; and lack of explanation concerning losses of participants at each stage. We did not classify any study as weak.

### Geographical Coverage

Researchers conducted the studies with human participants on three continents: Africa, Asia, and South America (see Table 1). We observed that all studies prioritized areas marked by (i) nutritional deficiencies at the population level and (ii) consumption of foods hunted and gathered in the context of local food systems. The samples ranged from *n* = 33 households (21) to *n* = 6,226 individuals (4).

Considering rural-urban classification, 73% of the studies reported that data collection was conducted in rural areas exclusively. Of the remaining 27%, there were 9% in urban and rural areas, 9% in urban and peri-urban areas, and others that did not report geographic coverage considering urbanization levels.

### Biodiversity Assessment in Food Consumption Studies

Authors analyzed a variety of biodiverse foods. Some categories mentioned overlap, such as wild foods and wild plants. Some may express the same type of food, but with different terminologies, such as traditional plants, indigenous vegetables, and indigenous food plants. Regardless, in the following synthesis, we preserved the terms used by the authors. The biodiverse foods assessed were: all food sources; whole diet, with a focus on animals (hunted and farm-raised) and other food items obtained in natural habitats through hunting or gathering; animals; food plants; forest plants; cultivated plants; traditional plants; wild plants; indigenous vegetables; indigenous food plants; wild foods; and finally, fruits and vegetables produced in home gardens.

In general, studies showed that dietary diversity is positively associated with the consumption of wild plants (20, 21, 34). Studies have also shown that the greater the dietary diversity, the greater the adequacy of micronutrients consumed by a given human group (4, 14, 15, 25–27). Agricultural biodiversity within farms and home gardens was also associated with the nutritional quality of diets (29, 30, 32, 35) and with the livelihood potential of families (33). However, despite the richness of local food biodiversity, nutrient intake levels in several cases in rural areas were below recommendations, and food insecurity was also recurrent (12, 22, 24, 28). Some authors suggest that the socio-economic context (e.g., poverty, lack of education) undermines the capacity of some communities to recognize and exploit natural resources properly (12). Concerning urban areas, some authors argue that traditional establishments (e.g., open-air markets, street food markets) are essential to maintaining diversity and, consequently, minimal dietary adequacy (36).

#### Methods Used in the Assessment of Biodiverse Foods in Food Consumption Studies

Table 2 summarizes the main methods used in food consumption studies to address food biodiversity.

**Table 2:** Summary of methods applied to biodiversity assessment in food consumption studies.

**Type of method**	**Specifications**	**Technique**	**Number of the study**
Method for mapping biodiverse foods	Before dietary survey	Developing previous ethnographic assessment using different techniques to gather data, e.g., interviews with key informants, focus groups, local market surveys, free listing	#8 #9 #10 #14 #16 #21
		Collecting samples of local foods to identify with the help of herbariums and animal collections at universities and research institutes	#8 #9 #10 #21
	During dietary survey	Collecting samples of local foods to identify with the help of herbariums and animal collections at universities and research institutes	#2 #6 #7 #19
		Photographing samples	#2 #7
		Producing diaries with lists of local edible species	#3
		Focus groups discussions	#1 #2 #4 #7 #17
		Conducting interviews with key informants among community members and with agricultural technicians	#1 #2 #3 #7 #22
		Performing market research	#18 #22
		Counting with the support of a taxonomist	#3
		Using a field guide	#6
	After dietary survey	Consultation of the literature, collections, and lists of plants commonly available in the region	#2 #5 #6 #17 #19 #20
Method for assessing food consumption	Retrospective direct methods	Food frequency questionnaire (FFQ)	#4 #8 #9 #10 #19
		24-h recall	#1 #2 #4 #5 #6 #7 #8 #9 #10 #13 #14 #15 #16 #18 #19 #20 #21 #22
	Prospective direct methods	Weighed food records	#11
	Other methods	Semi-directed interview or questionnaire	#3 #17
		Direct observation	#3 #6 #14 #17
		7-day recall	#12

As Table 2 demonstrates, some researchers mapped local biodiversity before conducting dietary survey fieldwork, by using various ethnographic approaches and by collecting samples of local foods to identify with the help of herbariums and animal collections at universities and research institutes. During the dietary survey in the field, researchers used the following methods to identify species: collecting samples of local foods to identify a posteriori; photographing samples; keeping diaries with lists of local edible species; running focus group discussions; conducting interviews with key informants among community members and with agricultural technicians; performing market research; counting with the support of a taxonomist; and using a field guide. After fieldwork, consulting literature, collections, and lists of plants commonly available in the region was the only method applied.

The technique most used to map local food biodiversity, in 37% of the studies, was the collection of samples before or during the dietary survey fieldwork to identify resources a posteriori. Tied in second place were pre-field qualitative research and post-field consultation (literature, collections, and lists), present in 27% of the studies. Next, focus group discussion and interviews with key local informants, both during the dietary survey stage, were present in 23% of the studies.

Concerning taxonomic classification, 45% of the studies did not mention having addressed it and did not justify its absence. Among them, one used secondary data previously classified (4), and another highlighted the lack of taxonomic classification as a weakness of the investigation (22).

Retrospective direct methods were the most used by researchers. For example, the 24-h recall appeared in 82% of the studies, followed by the Food Frequency Questionnaire (FFQ), present in 23% of the studies (see Table 2). Among prospective direct methods, one study used weighed food records (28).

Still concerning methods, we identified weaknesses before, during, and after fieldwork. In the pre-fieldwork, the absence of taxonomic classification was the major limitation, but it was not reported by the majority of studies. During fieldwork, we identified several crucial limitations. Broegaard et al. (21) noted that, with direct observation, participants would intentionally fail to report all food products consumed due to legal constraints in protected areas. Furthermore, the authors recognized that the lack of repeated dietary assessments (e.g., 24-h recall and FFQ) hampered the analysis of within-person variability and estimation of usual dietary intake (4). After the dietary survey, the most frequent limitation was the lack of nutritional data for particular species in food composition tables (4, 20–22, 26, 28, 30, 35). Faced with this gap, Ghosh-Jerath et al. (25) ran food composition analyses of species that did not have data available. Due to budget constraints, other researchers analyzed diets by considering data of similar species (i.e., food matching) (35).

#### Indicators Employed to Measure the Contribution of Biodiversity to Nutrition

In Table 3, we summarize the indicators that enabled the assessment of food biodiversity in the reviewed studies. In total, authors mentioned 23 indicators.

**Table 3:** Summary of indicators employed to measure the contribution of biodiversity to nutrition in food consumption studies.

**Level of analysis**	**Indicator**	**General overview of the indicator accordingly to descriptions provided by the authors**	**Number of the study**
Production, collection, or supply	Crop species richness (CSR)	Number of species cultivated in a given area	#12
	Crop varietal richness (CVR)	Number of intraspecies varieties cultivated in a given area	#12
	Food accessed diversity index (FADI)	FADI is a ratio that expresses the productive capacity of the household in terms relative to the community. It is calculated by dividing the total number of edible resources grown, collected, accessed, and raised in a given family (*n*) by the maximum number of foods grown, collected, accessed, and raised in a given community (*N*). The quotient result is squared. Foods accessed from the market are not included in this index. Values closer to 0 indicate inequality in food access; closer to 1, the opposite	#10
	Plant genetic diversity (PGD)—cultivar	Number of cultivars available within a given species in the analyzed area	#14
	Plant genetic diversity (PGD)—germplasm	PGD (germplasm) is an indicator that expresses the degree of modification of a particular cultivar: (1) modern or high yield; (2) locally improved; (3) traditional or rustic; and (4) unknown. The use of this indicator requires support from agricultural experts	#14
	Species richness (SR)	Total number of species found in a sample	#6 #15
	Shannon-Wiener diversity index (H′)	H′ is an indicator that expresses the species diversity of a given sample. This indicator considers the number of species (how many species) and also the distribution among individuals considering richness, divergence, or evenness	#6 #17
Food consumption by population or household	Household diet diversity scores (HHDDS) or Household dietary diversity score (HDDS)	HHDDS or HDDS indicates the ability of a population or household members to access some food groups in a given period. The indicator ranges from 0 to 12. The following 12 food groups are used to calculate the HDDS indicator: cereals; roots and tubers; vegetable; fruits; red meat, chicken and offal; eggs; fishes and seafood; pulses and nuts; milk and dairy products; oil and fat; sugar and honey; and miscellany	#1 #7 #11 #12 #20
	Food biodiversity score (FBS)	Number of different types of food consumed by household members	#18
	Food consumption score (FCS)	FCS is calculated with the consumption frequency of different food groups consumed by a household during the seven days preceding the survey. There are different weights to express the “nutritional density” for each food group that compose the FCS: cereals (2); roots and tubers (2); vegetables (1); fruits (1); red meat, chicken and giblets (4); eggs; fish and seafood (4); pulses and nuts (3); milk and dairy products (4); oils and fats (0.5); sugar and honey (0.5); and miscellany. The FCS ranges from 0 to 112; generally, scores ≤ 21 are considered poor, from 21.5 to 35 borderline, and >35, acceptable	#11
	Food variety score (FVS)	Number of different types of food consumed at home in seven days. The FVS is used as a specification of FCS, describing the foods consumed within one specified food group. Ex.: Cereals and wheat products: corn, rice, pasta, bread, cornflour	#7
	Forest food consumption score (FFCS)	Number of forest food items consumed at home in 7 days	#7
	Minimum diet diversity (MDD)	MDD indicates the proportion of children aged 6 to 23 months who consumed at least five food groups in the 24 h before the inquiry. The following food groups are considered: breast milk; grains, roots and tubers; pulses and nuts; dairy products; meat; eggs; fruits and vegetables rich in vitamin A; and other fruits and vegetables. MDD is a measure that expresses the quality of the diet, given its association with MAR	#15 #22
	Minimum dietary diversity for women (MDD-W) or minimum dietary diversity index for women (MDD-W)	MDD-W indicates the proportion of women ages 15–49 who consumed at least five WDDS food groups in the past 24 h. MDD-W informs the quality of diets of women of reproductive age due to its relationship with MAR	#5 #10 #11 #13 #19
Food consumption by individuals	Women dietary diversity score (WDDS)	WDDS is a count of the total number of food groups consumed by women of reproductive age. The following food groups are considered: (i) white grains, roots and tubers, and plantain; (ii) pulses; (iii) nuts and seeds; (iv) dairy products; (v) meat, poultry, and fish; (vi) eggs; (vii) vegetables with dark green leaves; (viii) other vegetables and fruits rich in vitamin A; (ix) other vegetables; and (x) other fruits	#2 #13 #15
	Dietary species richness (DSR)	DSR corresponds to counting the number of different species consumed by the individual, per day. It is a measure that expresses the quality of the diet, given its association with MAR	#4 #13 #15 #19 #22
	Traditional food diversity score (TFDS)	DSR corresponds to counting the number of different local species (wild and cultivated) consumed by the individual, per day. It is a measure that expresses the quality of the diet, given its association with MAR	#19
	Simpson diversity index (D)	When applied to analyze diets, D indicates the number of different species consumed in a day and the distribution of the quantities consumed. To calculate D, the weight (in grams) of a given species consumed is divided by the total weight of all species consumed per individual per day	#15
Dietary consumption by individuals	Functional diversity (FD)	FD reflects the diversity of the nutritional composition of the species consumed by each individual. To calculate the FD it is necessary to have the nutritional composition data of all foods eaten in a day	#15 #20
	Protein intake from the consumption of wild animals	Percentage of contribution of a given food (or foods) to the total of protein intake	#3
	Contribution to total energy intake (%)	Percentage of contribution of a given food (or foods) to the total of energy intake	#21
	Nutrient adequacy ratio (NAR) or probability of adequacy (PA) or contribution to the intake of a given nutrient (%)	This indicator expresses the ratio between the nutrient intake of one individual and the dietary reference intake (i.e., RDA or EAR)	#2 #4 #7 #8 #9 #13 #16 #19 #21 #22
	Mean adequacy ratio (MAR) or mean probability of adequacy (MPA)	MAR is the arithmetic mean of the probable nutrient adequacy values (e.g., NAR) consumed by one individual in a day. It comprises the sum of all adequacy percentages divided by the number of nutrients evaluated. Thus, higher MAR values correspond to a greater diet adherence to nutritional requirements.	#13 #15 #16 #19 #22

At the first level of analysis, “Production, collection, or supply,” Species Richness (SR) and Shannon-Wiener Diversity Index (H′) were the indicators most used, comprising 9% of the studies. Regarding the second level, “Food Consumption by Population or Household,” we found that Household Diet Diversity Scores (HHDDS), also known as Household Dietary Diversity Score (HDDS), and Minimum Dietary Diversity for Women (MDD-W) were the most frequently used, one or both of which appeared in 22% of the studies. At the third level, “Food Consumption by Individuals,” Dietary Species Richness (DSR) was used in 18% of the studies. Finally, at the fourth level, “Dietary Consumption by Individuals,” Nutrient Adequacy Ratio (NAR) appeared in 45% of the studies, being the indicator most used within this category and among all indicators.

Even if we have described in Table 1 food security indicators, we do not present them in Table 3 or discuss them because they do not directly address food consumption of edible resources as a group or as species.

## Discussion

Our main objective with this review was to identify methods and indicators most used in food consumption studies to date. Based on our analysis, we highlight the following aspects.

We found that some researchers used biodiversity mapping strategies based on ethnographic approaches before the dietary assessment (12, 25–27). The studies developed by these researchers portrayed the local availability of biodiverse foods more consistently, i.e., presenting lists with local edible species satisfactorily identified. A rapid ethnonutrition assessment before the dietary survey provides data about cultural variables that interfere when elaborating lists of local foods, e.g., food classifications, food processing, food perception, seasonality (11). During this pre-assessment stage, several data gathering techniques allow the researcher to build a broader perspective of the local diet, considering food at the system level. Some of the methods used in the studies we reviewed were: interviews, focus group discussions, local market surveys, and free listing. The guidelines to assess biodiverse foods in dietary surveys indicate these methods as pathways to map the edible resources and related cultural uses by a given population (3).

Taxonomic identification of resources consumed locally is a critical step to build robust dietary surveys. The scientific identification of species will allow us to know precisely to which species people refer when using a given vernacular name. This step is crucial since popular names vary enormously among and even within different communities. For example, we know that traditional human populations from the Brazilian Caatinga use the popular name *bredo* for three edible species of three different botanical genera: *Portulaca oleracea* L., *Amaranthus viridis* L., and *Talinum fruticosum* (L.) Juss. Similarly, we can have different popular names to refer to the same species, as is the case of *cumaru* and *amburana-açu*, both referring to *Amburana cearensis* (Allemão) A.C.Sm (37). In the study developed by Chyne et al. (22), during data gathering, the researchers noticed a vegetable called *jalynniar*, with consumption reported in 15 different villages, consumed in ~100 g portions. As the researchers did not perform scientific identification of this plant, they also did not have the means to evaluate its nutritional properties. Omissions of this type can lead to results that do not express the accurate dietary profile, leading to wrong conclusions about the role of local biodiversity to foster good nutrition. We think that a possible explanation for this kind of weakness is the academic background of the research team. For example, research teams without specialists in biological and environmental sciences failed more frequently to provide taxonomic identification. We came to this conclusion after verifying the academic background of the authors of all selected studies. We identified that the absence of taxonomic identification was more common among manuscripts produced by disciplinary teams from social sciences and health sciences. On the other hand, a group exclusively composed of biological and environmental sciences scholars failed to choose a proper dietary assessment tool to gather consumption data (33). Multidisciplinary teams that included professionals from nutrition, natural sciences, and social sciences designed more robust approaches, choosing adequate methods and indicators to assess food biodiversity within diets.

A practical and efficient approach to address species identification in food consumption studies is to produce, before the dietary survey, a photographic guide containing all foods of interest to the assessment (3). None of the studies that we analyzed reported having used photo guides for this purpose. In two papers (12, 15), authors used photo guides to assess portion sizes and kitchen utensils, which are essential tools in well-designed food consumption studies, but they do not assess biodiversity information. The practice of using photo guides containing biodiverse foods is still incipient, and their absence can compromise the accuracy of the analyses due to poor species identification. Jacob et al. (37) recently developed a photo guide to assess biodiverse foods plants in the Brazilian Caatinga biome. The development of this guide involved six steps comprising (i) elaboration of the list of species, (ii) selection and production of pictures, (iii) assessment of the first version of the guide by local experts, (iv) adjustment of content and design, (v) assessment of the guide by botanists, and (vi) processing of the final version. In this paper, the authors also present the REA, which stands for Rapid Ethnonutrition Assessment method. This method allows prototyping dietary assessments with high efficiency, considering time and budget constraints. The article published by the authors explains REA step-by-step, giving clear examples of how this method benefits research teams by amplifying the perception and control of cultural variables that interfere with food consumption.

Another challenge to overcome when mapping biodiverse foods is related to wild animals. Some authors described difficulties with underreporting in dietary inquiries due to legal restrictions regarding the consumption of these animals (14, 20). In these cases, when food consumption conflicts with legal rules and ethical limits, building a good rapport and trust are research practices that allow the interviewees to feel more inclined to report consumption (38–40). Another challenge reported by the authors of the manuscripts we analyzed was obtaining samples of wild animals to develop composition analyses. Dialogue with local authorities can be a strategy for accessing samples since dead animals sometimes are apprehended and discarded by these authorities.

Seasonality is another topic to consider when mapping foods available or when conducting dietary surveys. Reproduction cycles of animals, plants, algae, and fungi vary in different seasons. This factor is especially relevant in traditional food systems in rural areas, where diets tend to change with the season (41). We can approach seasonality by mapping biodiversity in different seasons, by running systematic reviews, and by consulting official data capable of informing edible biodiversity. Furthermore, capturing seasonal variation by dietary surveys is essential to estimate usual dietary intake (42). We can approach this task by conducting a repeated 24-h recall, by applying food record methods, and by using FFQ.

Even after overcoming barriers in the data gathering stage, composition data of biodiverse foods are frequently insufficient to perform a dietary analysis. Data in food composition tables often are limited because they include analysis of food available in markets and do not consider local crop varieties that vary in their nutrition content due to individual characteristics, climate, soil, etc. For instance, a recent study analyzing the nutrition content of millets showed that some varieties could have three times more iron than others (43). Therefore, using these varieties could be strategic to reduce iron deficiency anemia with low-cost potential. This example is beneficial to illustrate that the lack of local foods in food composition tables can lead to over- or underestimating energy, macro- and micronutrients, bioactive compounds, and anti-nutritional factors in dietary assessments. The problem of analysis gets even more serious due to the fact that some countries do not even have their own food composition tables, e.g., the Democratic Republic of Congo (12). Other countries neglect to include in their composition tables species used by traditional communities. For example, 80% of the wild species consumed by people in the Brazilian Caatinga are absent from food composition tables of Brazil (18). The lack of composition data sometimes makes the effort to gather consumption details fruitless since researchers will not have the means to interpret these data. When composition tables do not provide the data, and there is no means to analyze the food directly, Food Matching (FM) is the strategy recommended by the Food and Agriculture Organization of the United Nations (44). In FM, the researcher chooses the best substitute for the missing data in composition tables from other countries, scientific articles, theses, gray literature, and food labels. When the data is not available from any of these sources, the recommendation is to choose three similar food items present in composition tables and calculate the average of nutrients. This strategy has severe limitations, such as over- or underestimating nutrients and bioactive compounds content. However, it is an analytical solution in the complete absence of data.

Concerning the geographical coverage of assessments, we highlight that food consumption studies conducted in urban contexts present particular challenges to the approach of biodiversity. Some of these challenges are the higher degree of complexity of urban and peri-urban food systems and the higher contributions of processed foods to diets in these settings (4). It is urgent to find ways to overcome these limitations in order to better analyze the role of biodiversity in diets in these contexts since these settings concentrate several challenges, and several opportunities, in the implementation of the agenda of the sustainable development goal. So far, considering the available evidence, some authors show that consumption of diversified foods is more significant in urban areas than in rural and peri-urban areas due to a better supply and access to food (15, 23). However, we need to evaluate this information carefully. In some cases, a large number of species does not necessarily reflect a higher quality of diets. For example, the frequency of consumption of ultra-processed and industrialized foods in urban areas when compared to rural areas tends to be high (23). Conti et al. (15) also highlight that households in peri-urban regions tend to have more land available to establish home gardens when compared to those in urban areas, which can increase the consumption of *in natura* and minimally processed food by those households. Considering food selling points, Wertheim-Heck and Raneri (36), who conducted research in two low-income urban districts in Hanoi, Vietnam, reported that one of the biggest challenges in urban contexts is understanding the impact of types of food retailers on dietary diversity. They formulated a research experiment to assess this problem. To measure the consumption of biodiverse foods, they tried to use the Dietary Species Richness (DSR) indicator, but they could not apply it properly due to issues in the taxonomic identification of species. The complexities of biodiverse food consumption in urban and peri-urban contexts need to be better framed by researchers in order to guide policy-making that affects people living in these settings.

We believe that the DSR is the most promising indicator to evaluate the impact of biodiversity in diets because it provides a proxy to assess simultaneously diets and biodiversity conservation. Lachat et al. (4) highlight that, given the conflicts in reconciling environmental and nutrition policies, the DSR is a valuable tool that integrates biodiversity, food, and health. In addition, DSR provides more specific details at the species level than classic indicators that focus on food groups. This characteristic allows a more accurate analysis at the nutrient level. We also highlight the value of using agrobiodiversity indicators in food consumption studies because they can inform pathways to promote sustainable diets at the food systems level. We can use these indicators to test whether agricultural biodiversity is protective of nutrition. For example, using the Crop Species Richness (CSR) indicator, Jones (29) demonstrated a relationship between crop production and dietary diversity. Finally, to consider the genetic diversity of edible resources below the species level and its relationship with nutritional profile, the Plant Genetic Diversity (PGD) indicator provides a reference useful for understanding, protecting, and promoting genetic diversity (31).

This study has one limitation: the lack of specific protocols for assessing the overall quality of the studies. To address this limitation, we adapted a consolidated protocol.

## Conclusion

Our study summarizes methods and indicators that researchers use to address biodiversity in food consumption studies. We believe researchers in the future can avoid many of the limitations of current methods by ensuring that teams are interprofessional. In our opinion, one professional trained in food consumption studies, a person with a background in ethnographic methods, and a taxonomist are fundamental actors on a team. We emphasize that most of the indicators we summarized are not sensitive enough to biodiversity since they do not measure edible resources at the species level. This limitation creates information gaps, especially in the case of wild or neglected species, which hampers our ability to estimate the actual contribution of these foods within diets. In this sense, the DSR is promising, even if applying this indicator can be a real challenge due to the necessity to prospect the species considering their taxonomic classification. Finally, to correctly evaluate the role of biodiverse foods in diets, we need good composition data for these species or the resources to conduct composition analyses. Securing resources is a challenge due to the limited funding available. Many biodiversity hotspots are underdeveloped countries with limited resources to fund science and lack of scientific experts who are well-trained to conduct the well-designed studies systematically. By considering the strategic role of food biodiversity in transforming global food systems, especially in light of the environmental crisis we are currently facing worldwide, we suggest that international research agencies could apply more resources to scholars in the Global South working within the food biodiversity agenda.

## Data Availability Statement

The original contributions presented in the study are included in the article/Supplementary Material, further inquiries can be directed to the corresponding author/s.

## Author Contributions

MM, SS, and MJ analyzed and interpreted the data and were major contributors in writing the manuscript and responsible for all components of the research and manuscript. CT assisted in analyses, data interpretation, and in writing the first drafting and final version of the paper. SL and DM had substantially revised the first drafting and final version of the paper. All authors have made important contributions in the research conception and by reading and approving the final manuscript.

## Funding

This study was funded by the Federal University of Rio Grande do Norte through a Scientific Initiation research scholarship to MM (UFRN call 01/2019). This funding source had no role in the design of this study nor in its execution, interpretation of data, analyses, or decision to submit results. The Brazilian Coordenação de Aperfeiçoamento de Pessoal de Nível Superior financed the fee to publish this article (Finance Code 001).

## Conflict of Interest

The authors declare that the research was conducted in the absence of any commercial or financial relationships that could be construed as a potential conflict of interest.

## Publisher's Note

All claims expressed in this article are solely those of the authors and do not necessarily represent those of their affiliated organizations, or those of the publisher, the editors and the reviewers. Any product that may be evaluated in this article, or claim that may be made by its manufacturer, is not guaranteed or endorsed by the publisher.
