# Supplementary material for: Assessment of Biodiversity in Food Consumption Studies: A Systematic Review
Source: Front Nutr. 2022 Jun 14;9:832288. doi: 10.3389/fnut.2022.832288 (PMC9237621; doi:10.3389/fnut.2022.832288)
Supplement: Supplementary file 2 [file Data_Sheet_2.pdf]

Supporting Information File 2. Research strategy for systematic review

Database	Strategy
SCOPUS (ELSEVIER)	((FOOD OR DIET OR NUTRITION [ARTICLE TITLE, ABSTRACT, KEYWORDS]))<br>AND<br>(("DIETARY ASSESSMENT" OR "FOOD CONSUMPTION" OR "DIETARY SURVEYS" OR "DIETARY INTAKE" [ARTICLE TITLE, ABSTRACT, KEYWORDS]))<br>AND<br>((BIODIVERSITY OR "SPECIES DIVERSITY" [ARTICLE TITLE, ABSTRACT, KEYWORDS]))<br>REFINE RESULTS: LIMIT TO (DOCUMENT TYPE: ARTICLE) (LANGUAGE: ENGLISH, PORTUGUESE, SPANISH)
MEDLINE/ PubMed	((FOOD OR DIET OR NUTRITION[MESH TERMS]))<br>AND<br>(("DIETARY ASSESSMENT" OR "FOOD CONSUMPTION" OR "DIETARY SURVEYS" OR "DIETARY INTAKE"[MESH TERMS]))<br>AND<br>(BIODIVERSITY OR "SPECIES DIVERSITY"[MESH TERMS]))<br>LANGUAGES: ENGLISH OR PORTUGUESE OR SPANISH
GOOGLE SCHOLAR	(("FOOD" "BIODIVERSITY" [COM TODAS AS PALAVRAS])<br>AND<br>("NUTRITIONAL QUALITY OF DIETS" [COM A FRASE EXATA]))<br>[EM QUALQUER LUGAR DO ARTIGO] [IDIOMAS: INGLÊS, PORTUGUÊS, ESPANHOL]
WEB OF SCIENCE	TÓPICO:<br>((FOOD OR DIET OR NUTRITION)<br>AND<br>("DIETARY ASSESSMENT" OR "FOOD CONSUMPTION" OR "DIETARY SURVEYS" OR "DIETARY INTAKE")<br>AND<br>(BIODIVERSITY OR "SPECIES DIVERSITY"))<br>TIPO DE DOCUMENTO: ARTIGO. IDIOMA: INGLÊS, PORTUGUÊS, ESPANHOL.
